# Genetic Variation in Taste Receptor Genes (*SCNN*1*B*, *TRPV*1) and Its Correlation with the Perception of Saltiness in Normotensive and Hypertensive Adults

**DOI:** 10.1155/2021/5559831

**Published:** 2021-06-04

**Authors:** Pradtana Tapanee, Diane K. Tidwell, M. Wes Schilling, Daniel G. Peterson, Terezie Tolar-Peterson

**Affiliations:** ^1^Department of Food Science, Nutrition, and Health Promotion, Mississippi State University, Starkville 39762, MS, USA; ^2^Institute for Genomics Biocomputing and Biotechnology, Mississippi State University, Starkville 39762, MS, USA

## Abstract

**Background:**

Different taste preferences correlated with genetic variations may lead to food consumption patterns that contribute to nutrient-related health outcomes such as hypertension.

**Objectives:**

The aim of this study was to determine whether single nucleotide polymorphisms (SNPs) in the salt taste receptor genes *SCNN*1*B* and *TRPV*1 affect salt taste perception among normotensive and hypertensive people.

**Materials and Methods:**

We conducted a cross-sectional case control study by design consisting of a normotensive and hypertensive group. Participants were 253 adults with age range of 20–82 residing in Mississippi, USA. For each of 128 normotensives and 125 hypertensives, the salt taste recognition threshold and salt taste receptor genotype were determined.

**Results:**

The hypertensive group had a higher salt taste recognition threshold than the normotensive group (*P* < 0.001). The polymorphism of *TRPV*1, rs4790522, with the AA genotype was associated with a higher salt recognition threshold (lower salt taste sensitivity) in people with hypertension and obesity. Moreover, the polymorphism of *TRPV*1, rs8065080, and *SCNN*1*B*, rs239345, genes were associated with a risk of hypertension (*P*=0.016 and *P*=0.024).

**Conclusion:**

Correlations between SNPs, salt taste sensitivity, and hypertension risk were observed. People with hypertension had a higher salt taste threshold than those with normotension.

## 1. Introduction

Taste preference is influenced by environmental and genetic factors [1]. Human taste perception develops in childhood and has been emphasized as a driver of food preference [2]. Previous research reported correlations between salt preference with shorter stature and a higher percentage of body fat in children between 5 and10 years of age [3]. Salt taste perception impacts food preference and sodium consumption [4]. Two membrane receptor/channel proteins, ENaC (epithelial sodium channel, formed by the gene products of *SCNN*1*A, SCNN*1*B, SCNN*1*G*, and *SCNN*1*D*) and *TRPV*1 (transient receptor potential cation channel subfamily V member 1, encoded by the *TRPV1* gene), have been identified as salt taste receptors. Two single nucleotide polymorphisms (SNPs), one in the gene *SNN*1*B* and one in *TRPV*1, have been correlated with salt taste sensitivity [5]. Clinical trials confirmed positive effects of dietary sodium reduction on blood pressure and a decreased incidence of stroke and mortality due to coronary heart disease [6, 7]. However, it is unclear if there is an association between salt taste threshold and hypertension status [8, 9]. No research has been conducted to examine a possible association between salt taste perception and taste receptors in hypertensive patients. The main objective of this research was to determine whether SNPs in the salt taste receptor genes (*SCNN*1*B, TRPV*1) affect salt taste perception in hypertensive versus normotensive participants.

## 2. Materials and Methods

### 2.1. Participants

This cross-sectional study included 253 adults that were between 20 and 82 years of age. The study was conducted over 14 months, from February 2019 to April 2020. The participants were recruited from the community at health-related events, conferences, community centers, or churches in Mississippi. Posters and emails were distributed that announced the research project. All participants were categorized either in the hypertensive or normotensive group. Inclusion criteria for the hypertensive group included being 20 years of age or older, being diagnosed with hypertension, and being on a stable regimen of antihypertensive therapy for at least one month. Normotensive participants were volunteers aged 20 years or older and never diagnosed with hypertension. To obtain a 95% confidence level at 80% power [10], we determined that we needed a total of 125 participants in both the normotensive hypertensive groups. Participants in both groups had to be willing and able to complete all parts of the study, read and write in English so as to understand the consent form (in English), and provide a saliva sample. Exclusion criteria for both groups included any history or evidence on physical examination of taste impairment, xerostomia (dry mouth), regularly taking medication affecting taste or saliva production, or confirmed pregnancy. The study was approved by the Institutional Review Board (IRB) at Mississippi State University (IRB-18-510), and all procedures were in accordance with the ethical standards of the IRB and the Helsinki Declaration of 1975, as revised in 2000. All participants provided informed consent prior to participation.

### 2.2. Saliva Collection and Genotyping

In this cross-sectional study, participants were asked to provide two milliliters of saliva using the passive drool collection method with the Saliva Collection Aid from Salimetrics®. Saliva samples were blotted onto sterile filter paper (Fisher Scientific, Pittsburgh, PA). A drying board was used for saliva filter paper blots. DNA was extracted from the filter paper with dried saliva using the TaqMan Sample-to-SNP^TM^ Kit (Applied Biosystems, Foster City, CA). Four SNPs—specifically, *SCNN1B* gene: rs239345 and rs3785368, and *TRPV1* gene: rs8065080 and rs4790522—were analyzed using TaqMan allelic discrimination assays and the QuantStudio5 real-time PCR system. All alleles are reported in the forward orientation. The probe sequences from 5′-3′for the genotyped SNPs are represented in Table 1.

### 2.3. Salt Taste Perception

Salt taste perception was determined using a modified Harris–Kalmus procedure [11]. The lowest concentration that participants could recognize of the salty taste in the salt solution series was defined as the recognition threshold. All samples were presented at the same time and participants were asked to identify the quality of the taste after holding the sample in their mouth for at least 5 seconds using the sip-and-spit procedure. Participants tasted the solution of sodium chloride (NaCl) (Morton Salt, noniodized salt, Chicago, Illinois, USA) one by one in ascending order of concentrations (0.000, 0.001, 0.002, 0.004, 0.008, 0.016, 0.032, 0.064, 0.128, and 0.256 mol/L) and identified which sample had a salty taste. One sample at the concentration, which they recognized with 2 blank samples, was sorted into “salty taste” and “no taste or water.” Failure to sort the solutions correctly into two groups led to an increase of concentration until the recognition threshold was obtained.

### 2.4. Data Analysis

All data were analyzed using Statistical Package for Social Sciences (SPSS) version 24.0 (SPSS Inc., Chicago, Illinois, USA). Frequencies and percentages described participants' general characteristics and genotype variation between groups. Chi-squared test and Cramer's V test were used to analyze the differences in demographic variables between groups. The genotype frequencies of the four SNPs were tested for consistency with Hardy–Weinberg Equilibrium (HWE) by using chi-square tests. Mean differences in salt taste thresholds between and within hypertensive and normotensive participants were performed using analysis of variance (ANOVA). Allele frequencies in different groups of participants were compared using the chi-square test. Multiple linear regression models were used to assess the association of genotype and other confounding factors on the salt taste threshold. The criterion of significance was set at *α* = 0.05.

## 3. Results

The descriptive characteristics of 128 normotensive participants and 125 hypertensive participants are reported in Table 2. The mean age of the normotensive group was 30.7 ± 11.0 years, with age range between 20 and 68 years. The normotensive group consisted of 80 Caucasians (62%), 35 African Americans (27.1%), 11 Asians (8.5%), and three Hispanic/Latino (2.3%). The hypertensive group ranged between 22 and 82 years with a mean age 53.3 ± 12.8 years. The hypertensive participant group was 73.9% African American (*n* = 68), 23.9% Caucasian (*n* = 22), and 2.2% Asian (*n* = 2). Female participants accounted for 74.2% of normotensives and 69.6% of hypertensives. The mean systolic and diastolic blood pressures of the normotensive group were 125.6 ± 15.9 and 78.4 ± 11.0 mmHg, respectively, while mean systolic and diastolic blood pressures of the hypertensive group were 145.2 ± 21.2 and 89.0 ± 12.9 mmHg, respectively. Both systolic and diastolic blood pressures of the hypertensive group were significantly higher than the normotensive group (*P* < 0.001). The study indicated that 48.2% of all participants were obese (BMI ≥30 kg/m^2^). There was a significant difference in BMI between the normotensive (28.1 ± 7.7) and hypertensive (33.4 ± 8.3) group (*P* < 0.001).

Table 3 presents demographic information in relation to hypertension status. Female participants represented 74.2% of normotensives and 69.6% as hypertensives. However, these differences were not statistically significant (*P*=0.414). Moreover, there were significant differences in age, race, and BMI between normotensives and hypertensives (*P* < 0.001). However, significant differences in salt taste recognition threshold were not observed between genders, age groups, races, and BMIs within the normotensive group (*P*=0.361, *P*=0.355, *P*=0.636, and *P*=0.798, respectively) nor were differences in threshold observed within the hypertensive group (*P*=0.599, *P*=0.816, *P*=0.338, and *P*=0.289, respectively) (see Table 4).

The mean recognition thresholds of the normotensive and hypertensive groups were 0.022 ± 0.020 mol/L and 0.035 ± 0.031 mol/L, respectively. The highest percentage of hypertensive participants (43%) recognized the salty taste at 0.032 mol/L, while 37% of normotensive participants recognized the salty taste at 0.016 mol/L. The difference in the recognition threshold was significantly higher for the hypertensive group of participants (*P* < 0.001).

Table 5 presents the genotype and allele frequencies of the four SNPs (*SCNN*1*B*, rs239345, rs3785368; *TRPV*1, rs8065080, rs4790522). All alleles were in Hardy Weinberg Equilibrium (HWE) with *P* values greater or equal to 0.05. Risk for hypertension was observed in two polymorphisms, specifically *SCNN*1*B*, rs239345, and *TRPV*1, rs8065080. The risk of having hypertension among the TT genotype of *TRPV*1, rs8065080 gene was approximately two times higher than that of individuals possessing one or more C alleles (CC and CT) with a 95% confidence interval of 1.14–3.13 (*P*=0.016). In addition, risk for hypertension was observed between the TT genotype and carriers of the A allele (AT and AA) in SNP rs239345, *SCNN*1*B* gene with odds ratio 0.55 (*P*=0.024) and a 95% confidence interval of 0.34–0.91.

**Figure 1:**
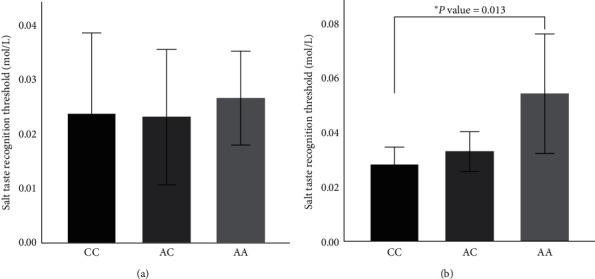
Salt taste recognition thresholds adjusted for BMI status and *TRPV1* genotypes for rs4790522 within the hypertensive group. Statistical differences were determined using One-way ANOVA, ^*∗*^post hoc test (*P* value <0.05). (a) Salt taste recognition threshold and *TRPV*1 genotype for rs4790522 in normal weight group. (b) Salt taste recognition threshold and *TRPV*1 genotype for rs4790522 in overweight/obese group.

Table 6 presents relationships between the salt recognition thresholds and other factors using stepwise multiple linear regression models. Factors that may affect salt recognition thresholds such as age, race, BMI, and SNPs were included in the models. The results showed only age (*B* = 0.254, *P*=0.004) was associated with the salt recognition thresholds in the normotensive group. In the hypertensive group, BMI (*B* = 0.208, *P*=0.017) and *TRPV*1 variant rs4790522 (*B* = 0.235, *P*=0.007) were associated with the salt recognition thresholds. After adjusting for BMI status in the hypertensive group, the result revealed that participants with *TRPV*1, rs4790522 with the AA genotype exhibited a higher salt taste recognition threshold than the CC homozygotes within the overweight/obese group (Figure 1).

## 4. Discussion

The salt results indicate that the hypertensive group had a higher salt taste recognition threshold than the normotensive group. This is in agreement with a study in Brazil which revealed that the salt taste recognition threshold for the normotensive group was 0.013 ± 0.017 mol/L, which was less than that of the hypertensive group, 0.027 ± 0.016 mol/L [13]. These researchers also indicated that the salt recognition threshold was positive and moderately correlated with total sodium intake for the whole group [13]. A study in the Turkish population reported that 55.5% of healthy participants recognized salt taste at a concentration of 0.016 mol/L [14]. They discussed that nutritional behavior might be influenced by cultural factors such as food introduction in childhood. In addition, a study in chronic kidney disease patients revealed that the salt taste recognition threshold was influenced by sodium intake and can be lowered by sodium restriction [15]. It was reported that diuretics and antihypertensive medication increased the gustatory threshold. This can possibly be explained by zinc deficiency due to increased zinc urinary excretion from the consumption of diuretics [15]. However, the mechanisms of zinc deficiency due to diuretics are unclear.

The risk of having hypertension among individuals having the TT genotype of *TRPV*1, rs8065080 was approximately two times higher than that of carriers of the C allele. In contrast, people with homozygous TT of *SCNN*1*B*, rs239345 had approximately 45% decreased risk of hypertension compared to carriers of the A allele. A study suggested that upregulating the protein expression of *SCNN*1*B* increased DNA methylation, which may lead to increased blood pressure [16]. *TRPV*1 protein, a gene product of *TRPV*1, may play an important role in the regulation of salt and water homeostasis and blood pressure [17, 18] since the degeneration of *TRPV*1-positive sensory nerves reduced salt sensitivity and increased blood pressure in a rat model [18]. This suggests that *TRPV*1 may play a counterbalancing role in inhibiting these prohypertensive systems [19].

In our study, there was a significant difference in age, race, and BMI between normotensive and hypertensive groups (*P* < 0.001) (Table 3). Previous research reported that age (≥50 years of age) and obesity have a significant effect on salt taste sensitivity [12, 20]. There was no significant difference in salt taste recognition threshold between gender, age groups, race, and BMI in both groups of our study (Table 4). However, the multiple linear regression model showed that age was associated with the salt recognition thresholds in the normotensive group, and in the hypertensive group, BMI and *TRPV*1 variant rs4790522 were associated with the salt recognition thresholds. Participants who were overweight/obese with *TRPV*1, rs4790522 with the AA genotype presented a higher salt taste recognition threshold. Interestingly, our study's findings are inconsistent with the results from the Chamoun et al. study, where children carriers of the C allele of rs4790522 had higher salt recognition thresholds than those with the A allele. The discrepancy suggests that age may have an effect on salt taste intensity. A previous study suggested that mutation of *TRPV*1 variant rs4790522, C allele, may affect the stability of the mRNA precursor to *TRPV*1 and decreased functionality of *TRPV*1 due to change to the miRNA binding site. Therefore, the decreased functionality of *TRPV*1 may affect the salt thresholds [21].

Although we did not observe significant differences in salt perception for *TRPV1*, rs8065080 genotypes, Dias et al. previously reported that carriers of the T allele had significantly lower salt perception threshold than CC homozygous participants for the C allele [5]. With regard to *SCNN*1*B*, rs239345 and *SCNN*1*B*, rs3785368, individuals with the AA genotype had significantly higher salt perception thresholds than those with other genotypes [5].

Our results of the correlation between genetic variation and salt taste threshold may help to develop an approach to prevent and manage hypertension. Current knowledge about risk factors for the development of hypertension does not include polymorphisms of taste receptor genes and their effect on the salt threshold. By identifying those polymorphisms as biomarkers for hypertension, we can not only identify individuals at risk and initiate prevention, but we can also understand and influence the dietary behavior of individuals who have already developed hypertension and develop targeted dietary therapies for these individuals.

### 4.1. Limitations of the Study

Strengths of our study include a sufficient sample size with a 95% confidence level at 80% power, the use of a reliable procedure to determine recognition thresholds, and a thorough and standardized genetic analysis. Although we reached the number of required participants, there were differences in age, BMI, ethnicity, and physical activity between normotensive and hypertensive groups. Nevertheless, there was no significant difference in salt taste recognition threshold between participants, all of whom were ≥20 years of age, within normotensive and hypertensive groups in this study. An additional limitation of the study is the lack of information on confounding variables such as smoking status and obesity [22].

## 5. Conclusions

The hypertensive group had a higher salt taste recognition threshold than the normotensive group. In addition, the polymorphism *TRPV*1, rs4790522 with AA genotype was associated with a higher salt recognition threshold (lower salt taste sensitivity) in people with hypertension and obesity. Participants that were homozygous TT for *TRPV*1, rs8065080 had approximately two times higher the risk of having hypertension than that of carriers of the C allele. In contrast, people with homozygous TT of *SCNN*1*B,* rs239345 were 45% less likely to have hypertension compared to carriers of the A allele.

## Figures and Tables

**Table 1:** The probe sequences from 5′-3′ for the genotyped SNPs.

SNPs number	Nucleotide sequences
rs239345-FAM probe	AGCTGTTCTTCCTTGCTTTGCTTCC**T**CAGCTCTTGTGGTAAAGCTGTTTGC
rs239345-VIC probe	AGCTGTTCTTCCTTGCTTTGCTTCC**A**CAGCTCTTGTGGTAAAGCTGTTTGC
rs3785368-FAM probe	AGATAGACAGATAGATAGCTAAAGA**A**GAGTTTATTAAATATTAACTCACAT
rs3785368-VIC probe	AGATAGACAGATAGATAGCTAAAGA**G**GAGTTTATTAAATATTAACTCACAT
rs8065080-FAM probe	GTGGAAAACCCGAACAAGAAGACGA**T**GTAGACAAACATGAAACGGCACAGG
rs8065080-VIC probe	GTGGAAAACCCGAACAAGAAGACGA**C**GTAGACAAACATGAAACGGCACAGG
rs4790522-FAM probe	TGTCCCAGTAGAGACTGACCATCCA**C**ACTGTTTAGTAAAGTGAGTAAAAAC
rs4790522-VIC probe	TGTCCCAGTAGAGACTGACCATCCA**A**ACTGTTTAGTAAAGTGAGTAAAAAC

**Table 2:** General characteristics of 253 adults with and without hypertension.

Variables	Normotensive (*n* = 128)	Hypertensive (*n* = 125)
Mean age (years)^a^	30.5 ± 11.0	52.5 ± 12.9

Gender (*N*, %)		
(i) Female	95 (74.2)	87 (69.6)
(ii) Male	33 (25.8)	38 (30.4)

Weight (kg)^a^	78.8 ± 23.8	94.5 ± 23.7
Height (cm)	167.2 ± 8.7	168.4 ± 9.6
BMI (kg/m^2^)^a^	28.1 ± 7.7	33.4 ± 8.3

Highest educational graduation (*N*, %)		
(i) Less than high school degree	1 (0.8)	15 (12.1)
(ii) High school degree or equivalent	15 (11.7)	27 (21.8)
(iii) Some college but no degree	35 (27.3)	28 (22.6)
(iv) Associate degree	14 (10.9)	11 (8.9)
(v) Bachelor degree	38 (29.7)	23 (18.5)
(vi) Graduate degree	25 (19.5)	20 (16.1)

Systolic blood pressure^a^	125.6 ± 15.9	145.2 ± 21.2
Diastolic blood pressure^a^	78.4 ± 11.0	89.0 ± 12.9

Data are presented as mean ± SD or *n* (%). ^a^Age, weight, and BMI differences were determined using independent samples *t*-test with *P* < 0.001.

**Table 3:** Demographic variables of 253 adults with and without hypertension.

Variables	All (*n* = 253)	Normotensive (*n* = 128)	Hypertensive (*n* = 125)	*P* value
*Gender*				0.414^a^
Male	71 (28.1)	33 (25.8)	38 (30.4)	
Female	182 (71.9)	95 (74.2)	87 (69.6)	

*Age group* ^**b**^				<0.001^a^
20–50 age	168 (66.4)	118 (92.2)	50 (40.0)	
51 and older age	85 (33.6)	10 (7.8)	75 (60.0)	

*Race*				<0.001^c^
Non-hispanic white	107 (42.3)	80 (62.5)	27 (21.6)	
Black/African American	128 (50.6)	34 (26.6)	94 (75.2)	
Asian	14 (5.5)	11 (8.6)	3 (2.4)	
Hispanic/Latino	4 (1.6)	3 (2.3)	1 (0.8)	

*BMI*				<0.001^c^
Underweight	9 (3.5)	7 (5.5)	2 (1.6)	
Normal	70 (27.7)	52 (40.6)	18 (14.4)	
Overweight	52 (20.6)	25 (19.5)	27 (21.6)	
Obese	122 (48.2)	44 (34.4)	78 (62.4)	

Data are presented as *n* (%). BMI category using criteria of CDC (Centers for Disease Control and Prevention, 2014) for healthy adults. ^a^Chi-squared test. ^b^Age categories by a significant effect on salt taste sensitivity at 50 years or older [12]. ^c^Cramer's *V* test with *P* value <0.001.

**Table 4:** Salt recognition thresholds across gender, age, race, and BMI within normotensive and hypertensive groups.

Variables	Salt recognition thresholds (mol/L)	*P* value
Normotensives		
*Gender* ^a^		0.361
Male	0.0254 ± 0.020	
Female	0.0215 ± 0.020	

*Age group* ^ab^		0.355
20–50 age	0.0220 ± 0.019	
51 and older age	0.0284 ± 0.036	

*Race* ^c^		0.636
Non-hispanic white	0.0230 ± 0.019	
Black/African American	0.0222 ± 0.024	
Asian	0.0242 ± 0.022	
Hispanic/Latino	0.0073 ± 0.007	

*BMI* ^c^		0.798
Underweight	0.0209 ± 0.011	
Normal	0.0237 ± 0.022	
Overweight	0.0190 ± 0.019	
Obese	0.0235 ± 0.020	

Hypertensives		
*Gender* ^a^		0.599
Male	0.0324 ± 0.023	
Female	0.0357 ± 0.034	

*Age group* ^*ab*^		0.816
20–50 age	0.0355 ± 0.43	
51 and older age	0.0341 ± 0.21	

*Race* ^c^		0.338
Non-hispanic white	0.0279 ± 0.023	
Black/African American	0.0374 ± 0.033	
Asian	0.0160 ± 0.014	
Hispanic/Latino	0.0160 ± 0.000	

*BMI* ^c^		0.289
Underweight	0.0240 ± 0.011	
Normal	0.0244 ± 0.014	
Overweight	0.0308 ± 0.020	
Obese	0.0386 ± 0.037	

Data are presented as mean ± standard deviation. ^a^Statistical differences were independent samples *t*-test. ^b^Age categories by a significant effect on salt taste sensitivity at 50 years or older [12]. ^c^Statistical differences were determined using one-way ANOVA.

**Table 5:** Genotypes and allele distribution in normotensive and hypertensive groups.

Gene	Variant	Genotype	*N* (%)	MAF	HWE *P* value^a^
*Normotensive group*					
*SCNN*1*B*	rs239345	AA	6 (4.7)	0.24	0.537
		AT	49 (38.3)		
		TT	73 (57.0)		
	rs3785368	AA	4 (3.1)	0.16	0.557
		AG	32 (25.0)		
		GG	92 (71.9)		

*TRPV*1	rs8065080	CC	17 (13.3)	0.32	0.153
		CT	49 (38.3)		
		TT	62 (48.4)		
	rs4790522	AA	23 (18.0)	0.43	0.819
		AC	64 (50.0)		
		CC	41 (32.0)		

*Hypertensive group*					
*SCNN*1*B*	rs239345	AA	15 (12.0)	0.35	0.957
		AT	57 (45.6)		
		TT	53 (42.4)		
	rs3785368	AA	3 (2.4)	0.12	0.250
		AG	23 (18.4)		
		GG	99 (79.2)		

*TRPV1*	rs8065080	CC	9 (7.2)	0.22	0.094
		CT	36 (28.8)		
		TT	80 (64.0)		
	rs4790522	AA	30 (24.0)	0.49	0.926
		AC	63 (50.4)		
		CC	32 (25.6)		

HWE: Hardy–Weinberg Equilibrium, MAF: minor allele frequency. ^a^Chi-square test.

**Table 6:** Variables associated with salt recognition thresholds among normotensive and hypertensive groups using the multiple linear regression model.

Predictors	Normotensive group			Hypertensive group		
	*b*	Beta	*P* value^a^	*b*	Beta	*P* value^a^
Age	0.000	0.254	0.004^*∗*^	—	—	—
BMI	—	—	—	0.001	0.208	0.017^*∗*^
rs4790522	—	—	—	0.010	0.235	0.007^*∗*^

^a^Statistical significance was determined using stepwise multiple linear regression analyses, ^*∗*^*P* < 0.05.

## Data Availability

The data used in this study are available from the corresponding author upon reasonable request.
